# Pharmacokinetics of surotomycin from phase 1 single and multiple ascending dose studies in healthy volunteers

**DOI:** 10.1186/s40360-017-0123-z

**Published:** 2017-03-28

**Authors:** Gurudatt Chandorkar, Qiao Zhan, Julie Donovan, Shruta Rege, Hernando Patino

**Affiliations:** 1 Merck & Co., Inc., Kenilworth, NJ USA; 2 Janssen Research and Development, Child Health Innovation Leadership Department - CHILD, 920 Route 202 South, Raritan, NJ 08869 USA

**Keywords:** *Clostridium difficile*, *Clostridium difficile*-associated diarrhea, *Clostridium difficile* infection, Surotomycin

## Abstract

**Background:**

Surotomycin, a novel, orally administered, cyclic, lipopeptide antibacterial in development for the treatment of *Clostridium difficile*-associated diarrhea, has demonstrated minimal intestinal absorption in animal models.

**Methods:**

Safety, tolerability, and plasma pharmacokinetics of single and multiple ascending oral doses (SAD/MAD) of surotomycin in healthy volunteers were characterized in two randomized, double-blind, placebo-controlled, phase 1 studies.

**Results:**

Participants were sequentially enrolled into one of four SAD (500, 1000, 2000, 4000 mg surotomycin) or three MAD (250, 500, 1000 mg surotomycin twice/day for 14 days) cohorts. Ten subjects were randomized 4:1 into each cohort to receive surotomycin or placebo. Surotomycin plasma concentrations rose as dose increased (maximum plasma concentration [C_max_]: 10.5, 21.5, 66.6, and 86.7 ng/mL). Systemic levels were generally low, with peak median surotomycin plasma concentrations observed 6–12 h after the first dose. In the MAD study, surotomycin plasma concentrations were higher on day 14 (C_max_: 25.5, 37.6, and 93.5 ng/mL) than on day 1 (C_max_: 6.8, 11.0, and 21.1 ng/mL for increasing doses), indicating accumulation. In the SAD study, <0.01% of the administered dose was recovered in urine. Mean surotomycin stool concentration from the 1000 mg MAD cohort was 6394 μg/g on day 5. Both cohorts were well tolerated with all adverse events reported as mild to moderate.

**Conclusion:**

Both SAD and MAD studies of surotomycin demonstrated minimal systemic exposure, with feces the primary route of elimination following oral administration; consistent with observations with similar compounds, such as fidaxomicin. Results of these phase 1 studies support the continued clinical development of surotomycin for the treatment of *Clostridium difficile*-associated diarrhea.

**Trial registration:**

NCT02835118 and NCT02835105. Retrospectively registered, July 13 2016.

## Background

*Clostridium difficile*-associated diarrhea (CDAD) is a key cause of hospital- and community-acquired diarrhea and is associated with longer length of hospital stay, increased medical costs, and high rates of morbidity and mortality [1–3]. Attributable healthcare costs of CDAD in the United States are estimated to be between $433 million and $797 million per year [4]. Over the past decade, the incidence and severity of CDAD has increased across the United States, Canada, and Europe [5–7].

Despite having a clinical response rate of ~73 to 85%, vancomycin and metronidazole treatments are associated with recurrent CDAD in up to 45% of patients [8–11]. Aggressive vancomycin and metronidazole treatment is also associated with disruption of the intestinal microbiota and can promote colonization by vancomycin-resistant enterococci, highlighting the need for novel treatment options [12, 13]. An ideal agent for the treatment of CDAD should be associated with low levels of systemic absorption, resulting in high concentrations of the drug in the colon, combined with a narrow spectrum of activity against *C. difficile* to limit its impact on the established intestinal microbiota.

Surotomycin (CB-183,315; MK-4261) is a novel, orally administered, cyclic, lipopeptide antibacterial currently in phase 3 development for the treatment of patients with CDAD [14]. Surotomycin has a fourfold greater in vitro potency than vancomycin against *C. difficile* (minimum inhibitory concentration at which 90% of the isolates were inhibited [MIC_90_] = 0.5 μg/mL vs 2.0 μg/mL) and other Gram-positive bacteria with minimal impact on the Gram-negative organisms of the intestinal microbiota [15, 16]. Surotomycin, given orally, has been shown to be highly effective against both initial and relapsing hamster CDAD, with potency similar to vancomycin [14]. Surotomycin has previously demonstrated minimal intestinal absorption (<1%) in rats and dogs (Yin N et al., ICAAC 2010, unpublished data). The objectives of these phase 1 studies were to characterize the safety, tolerability, and plasma pharmacokinetic (PK) profile of single and multiple ascending oral doses of surotomycin in healthy volunteers.

## Methods

### Study design and participants

Written informed consent was obtained from all participants, and the protocols were approved by the institutional review board of the study site (West Coast Clinical Trials, LLC, Cypress, CA, USA). These randomized, double-blind, placebo-controlled, phase 1 studies consisted of a single ascending dose (SAD) study (protocol number LCD-SAD-08-04, NCT02835105) and a multiple ascending dose (MAD) study (protocol number LCD-MAD-08-08, NCT02835118). Both studies were conducted in accordance with the ethical principles originating from the Declaration of Helsinki and its amendments, consistent with Good Clinical Practices and local regulatory requirements.

Male and female subjects aged 18–75 years were eligible for these studies if considered by the investigator to be in good health with unremarkable current and past medical history before the first day of study. Subjects were required to have no clinically significant abnormalities in prestudy physical examination, electrocardiogram (ECG), and laboratory evaluations. Subjects with findings outside of the normal range were included in the study only if these findings were deemed not clinically significant by the investigator or medical monitor. Subjects had no evidence of prior chronic gastrointestinal inflammatory disease.

Exclusion criteria included incidence of *C. difficile* disease within 1 year before study entry (SAD); prior exposure to surotomycin (MAD); known hypersensitivity to lipopeptide antibacterials; any comorbid disease judged by the investigator to be clinically significant; any concomitant medication, except low-dose aspirin, paracetamol, and multivitamins, in the 2 weeks before dosing (investigator- and medical monitor-approved concomitant medications were permitted in patients aged 49 years and above); or any antibiotic within 30 days before the first dose of the study drug. Women who were unwilling or unable to use an acceptable method to avoid pregnancy or who were pregnant or lactating during the conduct of the study and until 1 month after last surotomycin dose were excluded.

**Fig. 1:**
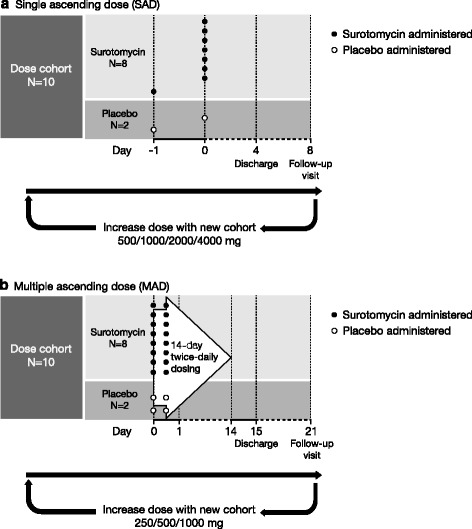
Single ascending dose (**a**) and multiple ascending dose (**b**) study overviews

For the SAD study, eligible subjects were sequentially enrolled into 1 of 4 dose cohorts: 500 mg, 1000 mg, 2000 mg, or 4000 mg surotomycin (Fig. 1a). In total, 10 subjects were intended to be randomized into each dose cohort in a 4:1 ratio to receive surotomycin (*n* = 8) or placebo (*n* = 2). At least 24 h before dosing the first full cohort, 2 subjects received surotomycin or placebo (randomized 1:1). The remaining 8 subjects were randomly assigned to surotomycin (*n* = 7) or placebo (*n* = 1) only if no significant safety findings or clinically significant abnormal laboratory values were reported for the first 2 subjects. Randomization was assigned by blinded study personnel and stratified by gender to achieve an equal number of male and female subjects in each cohort. Subjects aged 18–49 years and 50–75 years were equally distributed in each dosing cohort. Subjects received a single dose of surotomycin or placebo during the morning of day 1 (1 h after breakfast) and were followed as an inpatient through day 4 when they were discharged to return for a follow-up visit on day 8.

Eligible subjects were recruited and sequentially enrolled into 1 of 3 MAD dose cohorts: 250 mg, 500 mg, or 1000 mg surotomycin twice daily (BID) (Fig. 1b). A total of 10 subjects were randomized (4:1) to receive surotomycin (*n* = 8) or placebo (*n* = 2) in each cohort. Randomization was also stratified by gender and distributed by age to ensure that dosing cohorts were balanced. Subjects were dosed BID, once in the morning and once in the evening, for 14 consecutive days, with at least 8 ounces of water and approximately 1 h after breakfast and 1 h after dinner. Subjects were observed as inpatients through day 15 and were then discharged to return for follow-up on day 21.

In both studies, dose escalation to the next cohort occurred sequentially, and only after review of key safety data obtained from the previous cohort indicated that it was safe to proceed. The investigator and all personnel involved in the clinical or analytical evaluations of the study remained blinded to treatment until all cohorts had completed and the database was locked. Treatment doses were administered according to a randomization code by a pharmacist who was not an investigator or involved in study evaluations.

### Pharmacokinetic analysis

Any subject receiving at least one full dose of the study drug was included in the PK analysis population. PK analysis during the SAD study was conducted on serial plasma samples collected predose and at 30 min, 1, 2, 4, 6, 8, 12, 24, 48, and 72 h after dosing, and analyzed using a validated liquid chromatography-tandem mass spectrometry (LC/MS/MS) method, with a lower limit of quantification (LLOQ) of 1 ng/mL. Urine and stool samples were also collected during this period and analyzed using LC/MS/MS. Urine and feces were collected for 7 days after dose administration. The samples were analyzed using an API 5000 triple quadrupole mass spectrometer (Applied Biosystems/ScieEx, Concord, ON, Canada) using electrospray ionization in positive ion mode. Analyst^TM^ software (version 1.4.2., Applied Biosystems, Foster City, CA, USA) was used for data acquisition. In total, 8 calibration solutions with a range of 1.00 ng/mL to 1000 ng/mL were used as internal standards in addition to a blank. Inter-assay bias was determined to be –2.6 to 2.5% with inter-assay precision of 3.9 to 9.4%.

During the MAD study, serial plasma samples for PK analysis were collected predose and at 30 min, 3, 6, and 9 h after the morning dose on days 1 and 14, and before the morning dose on days 4, 7, 10, and 12 (trough levels). Stool was also collected in its entirety from all bowel movements for PK analysis following the morning dose on day 5 through predose day 6 in the 1000-mg BID dose cohort. Samples were analyzed using the same bioanalytical LC/MS/MS method as in the SAD study (LLOQ of 1 ng/mL).

Plasma PK parameters were calculated using standard noncompartmental methods in a validated version of WinNonlin (version 5.2, Pharsight, Mountain View, CA, USA). All concentrations that were below the LLOQ prior to the first detectable concentration were assigned a value of 0. All concentrations that were below the LLOQ after the first quantifiable concentration were designated as missing and replaced with a period. Actual sample collection times were used in the analysis of the concentration versus time profiles for individual subjects. Integration of plasma concentrations versus time was conducted using the linear-up, log-down function in WinNonlin. The following parameters were determined: maximum plasma concentration (C_max_), time of C_max_ (T_max_), area under the concentration-time curve (AUC) from 0 to last measurable plasma concentration (AUC_0-t_), AUC from 0 to infinity (AUC_0-∞_), percent of dose excreted in the urine, and terminal exponential half-life (t_½_).

Sample sizes were chosen based primarily on clinical considerations and were considered sufficient for the exploratory evaluation of single- and multiple-dose safety and PK.

### Safety analysis

Safety was monitored throughout the studies and on return for follow-up assessment on day 8 (SAD group) or day 21 (MAD group), by observation or reports of adverse events (AEs), and by changes in physical examination findings, vital signs, ECG, and laboratory tests. Concomitant medications and procedures were recorded. Any subject who received any dose of the study drug was included in the safety analysis population.

### Statistics

Statistical methods were primarily descriptive and no formal hypothesis tests were planned or completed. Data were summarized and analyzed using Statistical Analysis System SAS^®^ (version 9.1.3; SAS Institute, Cary, NC, USA).

## Results

### Subject demographics and characteristics

Both study groups were enrolled to completion with four 10-subject cohorts randomized in the SAD study and three cohorts of the same size randomized in the MAD study. All 40 subjects in the SAD group received all scheduled study medication. One subject (2000 mg) withdrew from the study early and missed the follow-up visit due to a family emergency. In all, 28 of 30 subjects completed the MAD study as planned. One subject (500 mg BID) discontinued treatment due to AEs of anxiety and dyspnea after 5 doses, and one subject (250 mg BID) did not complete the day-21 follow-up visit (considered lost to follow-up).

Subject demographics and baseline characteristics are summarized in Table 1. Half of all subjects enrolled in both studies were male, and treatment cohorts were generally well balanced with respect to age, race, and body mass index (BMI). In the SAD study, across all subjects who received surotomycin, the mean age was 42.9 years (range: 19 to 69 years) and mean BMI was 25.5 kg/m^2^. By comparison, subjects receiving placebo in the SAD study had a mean age of 28.3 years (range: 18 to 58 years) and a mean BMI of 23.9 kg/m^2^. For all subjects who received surotomycin in the MAD study, the mean age was 47.6 years (range: 20 to 70 years) and mean BMI was 27.4 kg/m^2^. Participants in the MAD study receiving placebo had a mean age of 48.8 years (range: 25 to 70 years) and a BMI of 25.8 kg/m^2^.

**Table 1:** Demographics and baseline characteristics

Characteristic	Single ascending dose						Multiple ascending dose				
	500 mg (*n* = 8)	1000 mg (*n* = 8)	2000 mg (*n* = 8)	4000 mg (*n* = 8)	Overall (*n* = 32)	Placebo (*n* = 8)	250 mg BID (*n* = 8)	500 mg BID (*n* = 8)	1000 mg BID (*n* = 8)	Overall (*n* = 24)	Placebo (*n* = 6)
Age (years)											
Mean ± SD	47.6 ± 14.94	42.0 ± 20.47	41.5 ± 15.58	40.6 ± 18.55	42.9 ± 16.89	28.3 ± 13.25	43.3 ± 10.43	47.9 ± 14.93	51.8 ± 13.51	47.6 ± 13.00	48.8 ± 20.91
Median	47.50	37.00	41.50	35.50	43.00	25.00	47.00	53.00	50.50	50.50	50.50
Min, max	23.0, 66.0	19.0, 67.0	21.0, 62.0	21.0, 69.0	19.0, 69.0	18.0, 58.0	27.0, 53.0	20.0, 69.0	29.0, 70.0	20.0, 70.0	25.0, 70.0
Sex, n (%)											
Male	4 (50.0)	4 (50.0)	4 (50.0)	4 (50.0)	16 (50.0)	4 (50.0)	4 (50.0)	4 (50.0)	4 (50.0)	12 (50.0)	3 (50.0)
Race, n (%)											
Asian	0	0	1 (12.5)	2 (25.0)	3 (9.4)	0	1 (12.5)	0	0	1 (4.2)	2 (33.3)
Black	2 (25.0)	2 (25.0)	1 (12.5)	0	5 (15.6)	2 (25.0)	1 (12.5)	2 (25.0)	2 (25.0)	5 (20.8)	1 (16.7)
White	6 (75.0)	5 (62.5)	6 (75.0)	6 (75.0)	23 (71.9)	6 (75.0)	6 (75.0)	6 (75.0)	6 (75.0)	18 (75.0)	3 (50.0)
Other	0	1 (12.5)	0	0	1 (3.1)	0	0	0	0	0	0
BMI (kg/m^2^)											
Mean ± SD	26.2 ± 4.11	24.0 ± 4.11	23.0 ± 2.83	28.7 ± 4.73	25.5 ± 4.41	23.9 ± 4.02	27.6 ± 1.28	27.8 ± 4.37	26.8 ± 3.15	27.4 ± 3.08	25.8 ± 4.48
Median	24.29	25.31	22.37	27.51	25.50	22.17	27.70	27.80	25.90	27.40	25.55
Min, max	22.7, 32.4	17.9, 28.3	19.8, 27.6	22.0, 25.3	17.9, 35.3	20.2, 32.2	25.5, 29.7	21.6, 35.2	23.6, 32.7	21.6, 35.2	19.8, 31.7

*BID* Twice daily, *BMI* Body mass index, *SD* Standard deviation

### Pharmacokinetic results

**Fig. 2:**
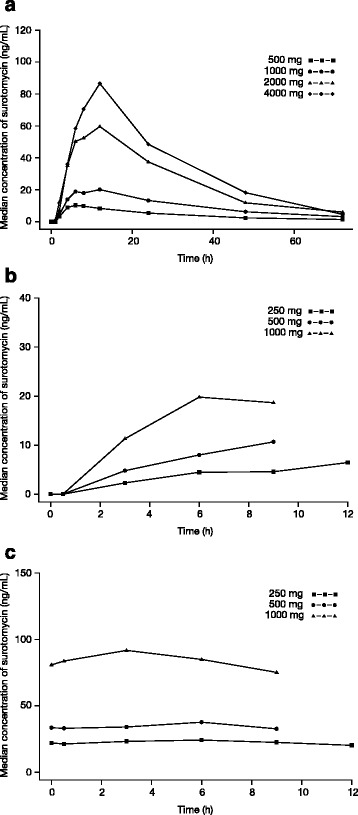
Median plasma concentration versus time profiles for surotomycin following administration of (**a**) a single dose, (**b**) the first dose on day 1, and (**c**) the morning dose on day 14

The median plasma concentration-time profiles of surotomycin following administration of single oral doses (SAD) and following administration of a single dose and repeated doses (MAD) are presented in Fig. 2. However, although quantifiable levels of surotomycin were observed in 4 subjects in the SAD 2 g cohort and 6 subjects in the 4 g cohort, a full plasma concentration-time curve was obtained in only 2 subjects, 1 in each dose cohort. Following a brief lag after the administration of a single dose, median plasma concentrations of surotomycin were quantifiable and seemed to increase with an increase in dose (Fig. 2a and b). Peak median plasma surotomycin concentrations were observed from 6 to 12 h after the first single dose in both the SAD and MAD groups. For patients receiving repeated surotomycin dosing, the median plasma concentration versus time profile on day 14 was flat (Fig. 2c), indicating that steady state had been reached and that the concentrations were essentially steady or constant during the dosing interval.

The PK parameters of surotomycin following administration of single oral doses (SAD) and a single oral dose and repeated oral doses (MAD) are summarized in Table 2.

**Table 2:** Summary of pharmacokinetic parameters for surotomycin. Following administration of a single dose and following administration of a single dose (day 1) and multiple doses (day 14)

	Single ascending dose				Multiple ascending dose					
					Day 1			Day 14		
Parameter	500 mg	1000 mg	2000 mg	4000 mg	250 mg BID	500 mg BID	1000 mg BID	250 mg BID	500 mg BID	1000 mg BID
T_max_, h (range)	6.0 (4.0–8.0)	10.0 (6.00–24.0)	8.0 (4.00–12.0)	12.0 (4.00–12.0)	9.0 (6.0–12.0)	6.0 (6.0–9.0)	7.5 (6.0–9.0)	4.5 (0.0–12.0)	6.0 (0.0–9.0)	1.8 (0.0–6.0)
C_max_, ng/mL (range)	10.5 (5.1–30.1)	21.5 (14.0–56.6)	66.6 (36.9–137)	86.7 (36.1–320)	6.8 (3.4–13.8)	11.0 (6.2–23.1)	21.0 (15.2–27.3)	25.5 (18.3–59.4)	37.6 (16.5–106)	93.5 (67.4–127)
AUC_0–t_, ng*h/mL (range)	260 (116–531)	664 (499–1648)	1863 (862–3284)	2481 (1419–6521)	41.2 (28.2–101.2)	49.4 (35.0–159)	118 (75.1–157)	274 (203–653)	313 (141–913)	771 (543–1029)
Half-life, h (range)	18.3 (15.5–22.8)	21.1 (14.7–32.5)	16.3 (11.4–34.5)	14.8 (10.4–21.5)	ND	ND	ND	ND	ND	ND
AUC_0-∞_, ng*h/mL (range)	317 (147–565)	702 (571–1754)	2085 (905–3355)	2572 (1507–6624)	ND	ND	ND	ND	ND	ND

*AUC* Area under the concentration-time curve, *AUC*_*0-t*_, *AUC* from 0 to last measurable plasma concentration; *AUC*_*0-∞*_, *AUC* from 0 to infinity; *BID* Twice daily, *C*_*max*_ Maximum plasma concentration, *ND* Not determined, *T*_*max*_ Time to maximum plasma concentration

**Fig. 3:**
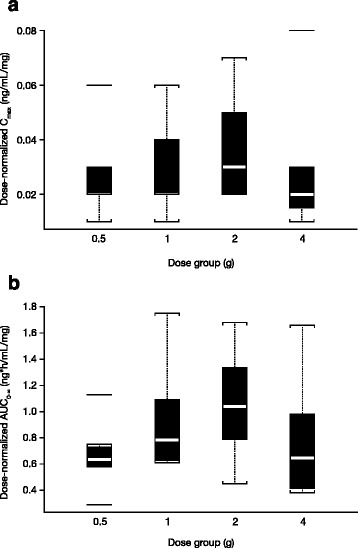
Dose-normalized (**a**) C_max_ and (**b**) AUC_0–∞_ of surotomycin following administration of single oral doses of surotomycin. AUC_0–∞_, area under the concentration-time curve from 0 to infinity C_max_, maximum plasma concentration

In the SAD group, median C_max_ ranged from 10.5 ng/mL in the 500-mg dose cohort to 86.7 ng/mL in the 4000-mg dose cohort, and the AUC_0-∞_ ranged from 317 ng*h/mL in the 500-mg dose cohort to 2572 ng*h/mL in the 4000-mg dose cohort. While the overall exposure of surotomycin as measured by C_max_ and AUC_0–∞_ increased with increases in dose, the median elimination half-life was independent of the dose administered and ranged between 14.8 and 21.1 h. In the MAD study, C_max_ on day 1 (following a single dose) was consistent with the findings of the SAD group and ranged from 6.8 ng/mL to 21.0 ng/mL. On day 14, the median C_max_ ranged from 25.5 ng/mL to 93.5 ng/mL, possibly suggesting an accumulation of surotomycin in the body following repeated dosing. No additional PK parameters for surotomycin could be computed due to the nature of the plasma concentration-time profile on days 1 and 14, and limited sampling. Figure 3 shows the dose-normalized C_max_ and AUC_0–∞_ for surotomycin after a single dose (SAD), indicating that surotomycin exposure increased in a dose-dependent manner.

In the SAD study, concentrations of surotomycin in the urine were detected in 12 of 32 subjects treated, and the median cumulative amount of the administered dose recovered in the urine was <0.01% over a 7-day period. The median cumulative fraction of the administered surotomycin dose excreted in the feces over a 4-day period ranged from 20.8 to 60.2% after a single dose (SAD). Stool levels of surotomycin increased proportionally with the dose administered. In the MAD study, the mean concentration of surotomycin from the 1000-mg BID dose cohort on day 5 was 6394 μg/g.

### Safety analysis

During the SAD study, a total of 13 (32.5%) subjects experienced at least one AE. Ten subjects had AEs that were assessed as treatment-related, including 9 (28.1%) of the 32 subjects who received surotomycin and 1 (12.5%) of the 8 subjects who received placebo. The most commonly reported treatment-related AE was diarrhea, reported in 5 (15.6%) surotomycin subjects and none of the placebo subjects (Table 3). Increased transaminases were reported in 2 (6.3%) of the 32 subjects who received surotomycin.

**Table 3:** Treatment-emergent AEs by system organ class and preferred term (safety population). Observed in ≥2 subjects in the a) SAD and b) MAD study

System organ class Preferred term	Single ascending dose				
	500 mg (*n* = 8) n (%)	1000 mg (*n* = 8) n (%)	2000 mg (*n* = 8) n (%)	4000 mg (*n* = 8) n (%)	Placebo (*n* = 8) n (%)
At least one TEAE	3 (37.5)	0	5 (62.5)	3 (37.5)	2 (25.0)
Gastrointestinal disorders	2 (25.0)	0	3 (37.5)	1 (12.5)	1 (12.5)
Diarrhea	1 (12.5)	0	3 (37.5)	1 (12.5)	0
Infections and infestations	0	0	0	2 (25.0)	0
Investigations	0	0	1 (12.5)	1 (12.5)	0
Transaminase increased	0	0	1 (12.5)	1 (12.5)	0
System organ class Preferred term	Multiple ascending dose				
	250 mg BID (*n* = 8) n (%)	500 mg BID (*n* = 8) n (%)	1000 mg BID (*n* = 8) n (%)	Placebo (*n* = 6) n (%)	
At least one TEAE	4 (50.0)	6 (75.0)	5 (62.5)	3 (50.0)	
Nervous system disorders	2 (25.0)	1 (12.5)	2 (25.0)	2 (33.3)	
Headache	1 (12.5)	1 (12.5)	0	2 (33.3)	
Gastrointestinal disorders	2 (25.0)	0	2 (25.0)	3 (50.0)	
Respiratory, thoracic, and mediastinal disorders	0	2 (25.0)	2 (25.0)	0	
Oropharyngeal pain	0	1 (12.5)	1 (12.5)	0	
General disorders and administration- site conditions	0	0	2 (25.0)	1 (16.7)	
Infections and infestations	0	1 (12.5)	1 (12.5)	0	
Musculoskeletal and connective tissue disorders	2 (25.0)	2 (25.0)	0	0	
Back pain	1 (12.5)	1 (12.5)	0	0	
Skin and subcutaneous tissue disorders	0	1 (12.5)	1 (12.5)	0	
Pruritus	0	1 (12.5)	1 (12.5)	0	

*BID* Twice daily, *MAD* multiple ascending oral dose; *SAD* single ascending dose; *TEAE* Treatment-emergent adverse advent

Although a total of 18 (60.0%) subjects experienced at least one AE during the MAD study, all reported AEs were considered unrelated to the study drug. The most common AE in the MAD group was headache, reported in 2 (8.3%) of the 24 subjects who received surotomycin (1 each in the 250-mg BID and 500-mg BID dose cohorts) and 2 (33.3%) of the 6 placebo subjects (Table 3). Constipation, back pain, oropharyngeal pain, and pruritus were each reported in 2 (8.3%) of the subjects who received surotomycin.

All reported AEs in both studies were mild to moderate in severity. No serious AEs were reported and none of the subjects discontinued the study due to AEs in the SAD group. One subject in the MAD group (500 mg BID) discontinued due to transient moderate anxiety and associated mild dyspnea. The events were reported 2 h after the 5^th^ dose of study treatment. The anxiety resolved within 4 h and the dyspnea within 3 min, both without the need for additional treatment.

## Discussion

In both SAD and MAD studies, C_max_ and AUC values were low, demonstrating the limited systemic exposure and that less than 0.01% of the administered compound was excreted in the urine. In addition to the clinical findings, complementary information obtained from preclinical animal studies with radiolabeled surotomycin suggests poor systemic absorption and exposure without elimination through the hepatobiliary route (Merck & Co., Inc., Kenilworth, NJ, USA, unpublished data). These results support the insignificant absorption of surotomycin observed in these clinical trials. Peak median surotomycin plasma concentrations were achieved between 6 and 12 h post-dose, also suggesting that a small amount is absorbed in the lower gastrointestinal tract in healthy volunteers.

In the SAD study, there appeared to be a degree of dose-non-linearity in surotomycin plasma concentrations, appearing to peak at the 2000-mg dose and decrease with the 4000-mg dose (Fig. 3). However, these data should be interpreted with care due to the very low concentrations detected in this study. In the SAD study at the 2000-mg and 4000-mg doses, a full plasma concentration-time curve could be determined from only two subjects. In addition, the concentrations detected were less than 3-fold of the LLOQ and thus no inferences could be made from these data.

As expected with a molecule with minimal absorption, stool levels of surotomycin increased proportionally with the dose administered. During the SAD and MAD studies, substantial concentrations of surotomycin were observed in the stool, indicating that this is the major route of elimination for orally dosed surotomycin. Assuming dose proportionality, stool concentrations of surotomycin should greatly exceed the MIC_90_ for *C. difficile*, suggesting that 250-mg BID oral dosing of surotomycin will be effective for the treatment of CDAD. Indeed, the low systemic exposure observed here did not come as much of a surprise, as it has been shown previously that in rats bioavailability after oral administration is very low (Yin N et al., ICAAC 2010, unpublished data).

Single oral doses of surotomycin (500 mg, 1000 mg, 2000 mg, or 4000 mg) were well tolerated, with all AEs reported as mild to moderate in severity. The most commonly reported AE in the SAD group was diarrhea, but this was not dose-dependent. Differences in AEs reported between the surotomycin cohorts and the placebo cohort may have been due to differences in the ages of subjects in the two groups (mean age ± standard deviation: surotomycin, 42.9 ± 16.89 and placebo, 28.3 ± 13.25), consistent with the observation that older populations have increased susceptibility to gastrointestinal complications of comorbid disease [17]. Multiple oral doses of surotomycin (250 mg, 500 mg, 1000 mg BID) were also well tolerated in healthy adult volunteers, with all AEs reported as mild to moderate in severity and no AEs reported as being related to the study drug.

## Conclusion

In summary, single (500 mg, 1000 mg, 2000 mg, 4000 mg) and multiple doses (250 mg, 500 mg, 1000 mg BID) of the novel lipopeptide antibiotic surotomycin demonstrated minimal systemic exposure, with the feces being the primary route of elimination following oral administration. These results are consistent with those observed with similar compounds, such as fidaxomicin. Results of these phase 1 studies support the continued clinical development of surotomycin for the treatment of CDAD. In addition to defining the efficacy profile of surotomycin against CDAD, currently being addressed in phase 3 trials, additional studies will determine the in vivo effects of surotomycin on intestinal microbiota.

## Abbreviations

AE: Adverse event
AUC: Area under the concentration-time curve
BID: Twice daily
BMI: Body mass index
CDAD: *Clostridium difficile*-associated diarrhea
C_max_: Maximum plasma concentration
ECG: Electrocardiogram
LC/MS/MS: Liquid chromatography-tandem mass spectrometry
LLOQ: Lower limit of quantification
MAD: Multiple ascending oral dose
MIC: Minimum inhibitory concentration
PK: Pharmacokinetic
SAD: Single ascending oral dose
